# Multilobed Osteochondroma of the Posterior Humeral Midshaft: A Rare Morphological Variant

**DOI:** 10.1155/cro/7763381

**Published:** 2026-07-18

**Authors:** Feras Abuqweider, Abdul Haq Mahmoud Shahin, Anas R. A. Bayyoud qasrawi, Ranya Abu Khalaf, Abdallah Badarna, Hanin Shatrit, Momen Sulyman, Mostafa Ibraheem, Qais Alnjoom

**Affiliations:** ^1^ Department of Orthopedics Surgery, Palestinian Red Crescent Society Hospital, Hebron, State of Palestine; ^2^ Department of Clinical Medical Sciences, Faculty of Medicine and Health Sciences, Palestine Polytechnic University, Hebron, State of Palestine, ppu.edu; ^3^ Palestinian Red Crescent Society Hospital, Hebron, State of Palestine; ^4^ Diagnostic Radiology Department, Al-Ahli Hospital, Hebron, State of Palestine, ahlihospital.com

**Keywords:** humerus, multilobed variant, orthopaedic surgery, osteochondroma, posterior midshaft, surgical excision

## Abstract

Osteochondroma, or osseocartilaginous exostosis, is the most common benign bone tumor, accounting for approximately 30%–35% of benign bone lesions and predominantly arising from the metaphyseal regions of long bones in young males. Mid‐diaphyseal origin and multilobed morphology are exceptionally uncommon and may present diagnostic and surgical challenges. We report a rare case of a solitary, multilobed osteochondroma arising from the posterior humeral midshaft in an 18‐year‐old male. The patient presented with a 1‐year history of a gradually enlarging, painless mass over the lateral aspect of the left upper arm, without functional limitation or neurovascular symptoms. Physical examination revealed a firm, immobile, nontender mass with intact distal neurovascular status. Magnetic resonance imaging demonstrated a pedunculated osseous lesion measuring 6.5 × 3.3 × 2.3 cm, exhibiting continuity with the humeral medullary cavity and a distinctive three‐lobed configuration, including a bilobed stalk. The cartilage cap thickness measured approximately 4 mm, with no surrounding soft tissue abnormalities. Surgical excision was performed via a posterior longitudinal approach, with careful identification and preservation of the radial nerve. Intraoperatively, three distinct pedunculated bony stalks arising from the posterior humeral midshaft were excised en bloc. Histopathological analysis confirmed a benign osteochondroma, showing mature trabecular bone capped by hyaline cartilage without atypia or malignant features. To the best of our knowledge, no previous report has specifically described this combination of posterior humeral mid‐diaphyseal location and multilobed morphology. This case expands the anatomical and morphological spectrum of osteochondroma and highlights the critical role of advanced imaging in preoperative planning, particularly for lesions in proximity to major neurovascular structures. Recognition of such atypical presentations is essential to ensure accurate diagnosis, safe surgical management, and avoidance of iatrogenic complications.

## 1. Introduction

Osteochondroma, also known as osseocartilaginous exostosis, is the most common benign bone tumor, accounting for approximately 30%–35% of all benign bone lesions, with a higher incidence in males [1]. It is characterized by a cartilage‐capped bony outgrowth that arises from the metaphysis of long bones, most commonly the femur, humerus, tibia, and pelvis [2].

Clinically, solitary osteochondromas are usually asymptomatic and present as firm, immobile, and nontender masses. However, when symptoms do occur, they may include localized pain, mechanical irritation, or impingement of surrounding soft tissues [3]. In some cases, the lesion can irritate adjacent structures, leading to the formation of a fluid‐filled bursa [4].

The growth of osteochondromas is typically slow and gradual, especially in skeletally immature individuals. Surgical excision may be considered if the lesion becomes symptomatic or shows signs of complications [5, 6]. Despite extensive literature on metaphyseal lesions, mid‐diaphyseal and multilobed variants remain exceedingly rare. To the best of our knowledge, no previous report has specifically described this combination of posterior humeral mid‐diaphyseal location and multilobed morphology [1].

## 2. Case Presentation

An 18‐year‐old male presented with a 1‐year history of a gradually enlarging, painless mass over the lateral aspect of the left upper arm. He denied any preceding trauma, constitutional symptoms, or limitation in joint movement.

On physical examination, a firm, nontender, immobile mass was palpated over the posterior midshaft of the humerus. The overlying skin was intact, and distal neurovascular function was preserved.

**Figure 1:**
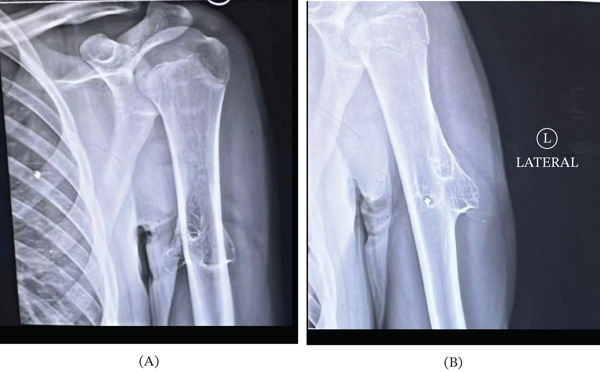
Plain radiographs of the left humerus demonstrating a multilobed osteochondroma arising from the posterior humeral midshaft. (A) Anteroposterior (AP) projection showing a lobulated osseous exostosis at the humeral midshaft with demonstrable continuity of the lesion with the underlying medullary cavity and native cortex. (B) Lateral projection demonstrating the posterior location of the lesion and its pedunculated morphology, with no evidence of cortical breach or aggressive periosteal reaction.

Plain radiographs of the left humerus, including anteroposterior (AP) and lateral projections, demonstrated a lobulated osseous exostosis arising from the posterior aspect of the humeral midshaft. The lesion exhibited cortical and medullary continuity with the parent bone, without evidence of cortical destruction, periosteal reaction, or soft tissue involvement. These findings are illustrated in Figure 1.

**Figure 2:**
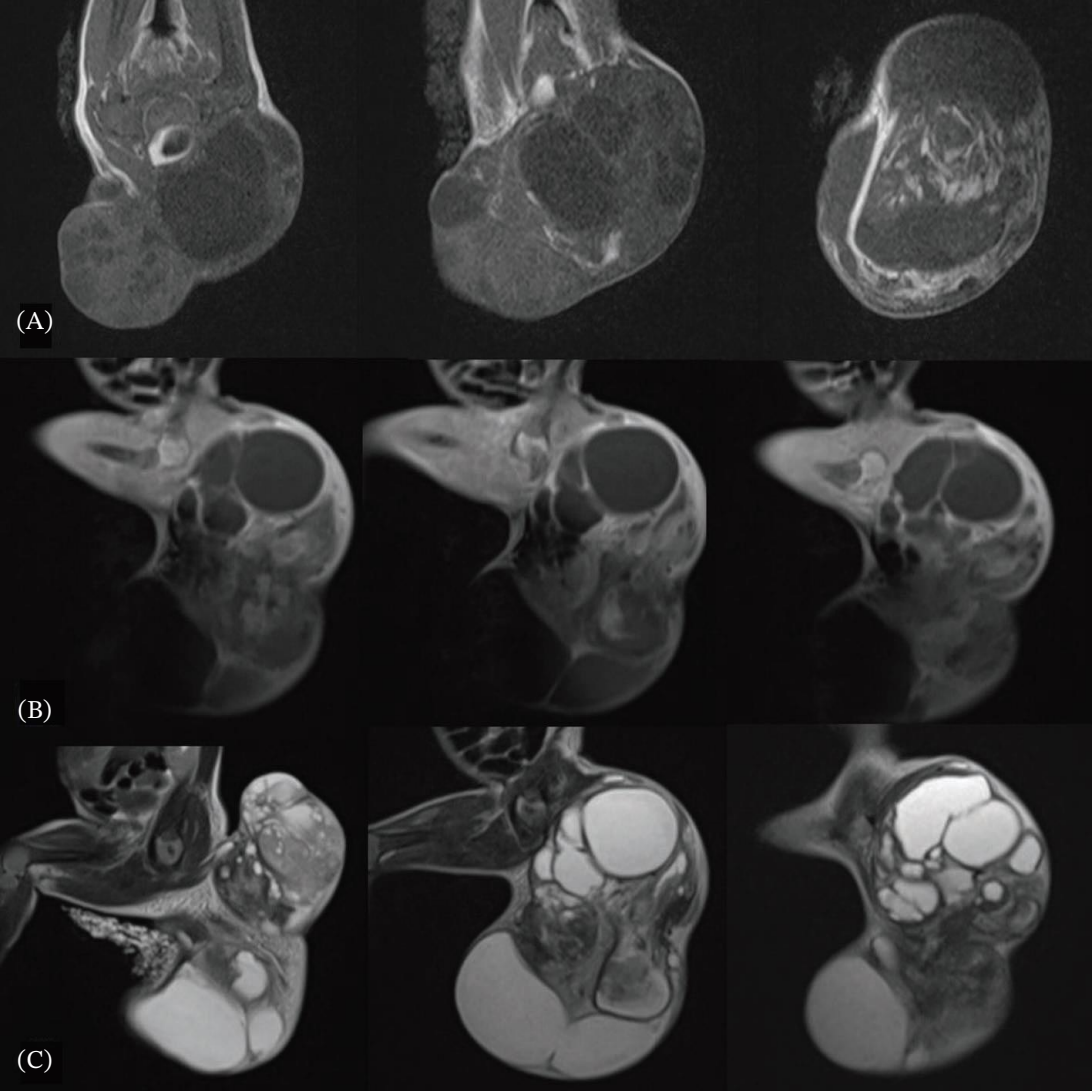
Multiplanar MRI of the left posterior humeral midshaft demonstrating a pedunculated osteochondroma. (A) T1‐weighted coronal image showing a lobulated osseous protuberance arising from the posterior aspect of the midshaft humerus, with continuity to the medullary cavity. (B) T2‐weighted coronal image highlighting the three‐lobed configuration of the lesion and the cartilage cap. (C) Fat‐suppressed axial T2‐weighted image demonstrating the cartilage cap thickness (~4 mm) and soft tissue interface.

Magnetic resonance imaging (MRI) of the left upper arm was performed using multiplanar, multisequence protocols without contrast enhancement. T1‐weighted and T2‐weighted coronal images, along with fat‐suppressed axial T2‐weighted sequences, revealed a pedunculated osseous lesion arising from the posterior midshaft of the humerus. The lesion measured approximately 6.5 × 3.3 × 2.3 cm in cephalocaudal, transverse, and AP dimensions, respectively. It demonstrated continuity with the underlying medullary cavity and exhibited a three‐lobed configuration, the largest lobe measuring 2.5 × 1.8 cm. The cartilage cap thickness was approximately 4 mm. No surrounding soft tissue abnormalities were noted. These findings are illustrated in Figure 2.

Surgical excision of the lesions at their bases was performed using standard osteotomes under general anesthesia via a posterior longitudinal approach. Although the patient reported no pain, functional limitation, or neurovascular deficit, the decision to proceed with surgery was based on the progressive enlargement of the lesion over 1 year, its atypical multilobed morphology warranting histopathological confirmation, and its proximity to the radial nerve, which posed a risk of future neurovascular compromise. Patient preference and cosmetic concern regarding the growing arm mass were also contributing factors. Dissection through the interval between the long and lateral heads of the triceps allowed exposure of the lesion. The radial nerve and adjacent neurovascular structures were identified and protected throughout the procedure.

**Figure 3:**
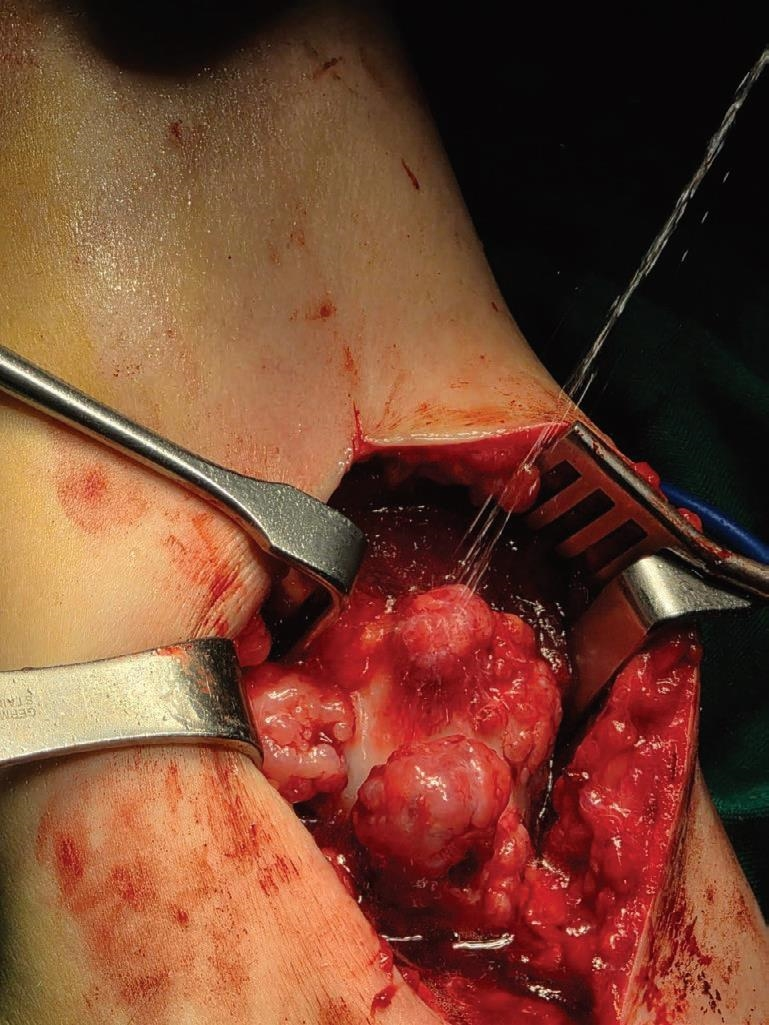
Intraoperative photograph of the exposed osteochondroma prior to excision. The image shows three distinct pedunculated bony stalks arising from the posterior humeral midshaft. One stalk demonstrates a bilobed morphology. The radial nerve and surrounding soft tissues were preserved during dissection.

Surgical exploration revealed three distinct pedunculated bony stalks arising from the posterior midshaft of the humerus. Notably, one of the stalks exhibited a bilobed morphology. All lesions were excised en bloc. Postoperative evaluation confirmed intact distal perfusion and prompt capillary refill, indicating preserved vascular integrity. At 6‐month follow‐up, the patient remained pain‐free with no evidence of local recurrence. Radial nerve function was fully preserved, with no postoperative motor or sensory deficit detected on clinical examination. The wound healed without complication, and no signs of infection, dehiscence, or seroma were observed. The patient reported no functional limitation, with full shoulder and elbow range of motion restored by the 6‐week postoperative visit and maintained at final follow‐up. The intraoperative appearance of the lesion is illustrated in Figure 3.

Histopathological examination of the excised specimen revealed a cartilage‐capped bony lesion composed of mature trabecular bone without evidence of cellular atypia, spindle cell proliferation, or mitotic activity. The findings were consistent with a benign osteochondroma.

## 3. Discussion

This case presents a rare form of osteochondroma arising from the posterior humeral midshaft in a young adult. To our knowledge, no previous reports have described a multilobed osteochondroma originating from the posterior humeral mid‐diaphysis. The lesion presented as a gradually enlarging painless mass without functional limitation or neurovascular symptoms and exhibited a distinctive multilobed morphology. Its proximity to the radial nerve necessitated meticulous preoperative imaging and precise intraoperative planning to prevent iatrogenic neurovascular injury. This report broadens the established morphological and anatomical spectrum of osteochondroma and underscores the clinical importance of recognizing atypical presentations.

Although osteochondromas typically arise from the metaphyseal regions of long bones, several rare reports have documented their occurrence in mid‐diaphyseal locations. Published cases include solitary lesions in the midshaft of the fibula [7], femur [8], and iliac bone [9], as well as secondary scalloping of the tibial midshaft due to adjacent fibular osteochondroma [10]. These cases collectively demonstrate that although midshaft involvement is anatomically possible, it remains an uncommon presentation.

In parallel, multilobed osteochondromas have been reported in small bones such as the hamate, where two distinct cases described lobulated growth causing mechanical symptoms or nerve compression [11, 12]. Additional reports have described multilobed lesions in the context of hereditary multiple exostoses (HME), particularly in the femur and scapula, where the lobulated architecture was attributed to aberrant growth patterns or mechanical stress [1]. In contrast to the lobulated lesions encountered in HME, the present patient exhibited a solitary, nonsyndromic lesion, underscoring a distinct morphological behavior. The current case further distinguishes itself by originating from the posterior humeral midshaft, a location not previously documented in the literature. The lesion exhibited three distinct lobes, including a bilobed stalk, and measured over 6 cm in length. Unlike other midshaft cases, which often presented with pain, deformity, or neurovascular compromise, this lesion presented as a gradually enlarging painless mass without functional limitation or neurovascular symptoms. Its posterior location posed unique surgical considerations, requiring careful dissection to preserve the radial nerve and surrounding structures.

Differential diagnosis included parosteal osteosarcoma, periosteal chondroma, and Nora′s lesion. The absence of cortical breach, marrow invasion, or cellular atypia on histology excluded these entities. Parosteal osteosarcoma was excluded by the absence of cortical destruction, permeative growth pattern, and soft tissue invasion on MRI, as well as the lack of nuclear atypia or atypical mitoses on histology. Periosteal chondroma was considered given the surface location, but was excluded by the demonstrable continuity of the lesion with both the cortex and medullary cavity, its large size, and the absence of a scalloped cortical erosion pattern. Nora′s lesion (bizarre parosteal osteochondromatous proliferation) was excluded by the characteristic zonal architecture of mature trabecular bone capped by hyaline cartilage without the cellular disorganization or “blue bone” matrix typical of that entity. The thin cartilage cap (~4 mm), cortical and medullary continuity, and uniformly benign histopathological appearance collectively confirmed the diagnosis of osteochondroma.

The combination of mid‐diaphyseal origin, posterior humeral location, and multilobed morphology distinguishes this case as a novel variant within the spectrum of osteochondroma presentations. It underscores the importance of advanced imaging in preoperative planning [3] and contributes to the growing recognition of atypical anatomical sites for benign bone tumors [7–10].

Multilobed osteochondroma of the posterior humeral midshaft represents a rare anatomical variant that poses both diagnostic and surgical challenges owing to its atypical location and morphology. Accurate preoperative imaging and complete surgical excision are paramount for achieving symptom resolution and minimizing the risk of recurrence. Heightened awareness of such atypical variants is essential to prevent iatrogenic radial nerve injury, ensure oncologically complete resection, and ultimately enhance clinical decision‐making and the accuracy of orthopedic literature. This report has inherent limitations as a single case report, which precludes generalization of findings. The follow‐up period of 6 months, although sufficient to confirm early outcomes, is relatively short; longer surveillance would be valuable to definitively exclude late recurrence and to further characterize the natural history of this morphological var.iant.

## Author Contributions

Feras Abuqweider performed the surgical procedure, supervised clinical management, and contributed to manuscript review and final approval. Abdul Haq Mahmoud Shahin assisted in surgical planning, coordinated clinical documentation, and contributed to literature integration. Anas R. A. Bayyoud qasrawi participated in postoperative follow‐up and assisted in manuscript drafting. Ranya Abu Khalaf and Abdallah Badarna conducted the literature review and contributed to manuscript formatting and reference verification. Hanin Shatrit interpreted radiological findings, selected imaging sequences, and prepared figure captions. Momen Sulyman and Mostafa Ibraheem supported data collection and assisted in organizing author affiliations and declarations. Qais Alnjoom structured the manuscript, harmonized all sections, and served as the corresponding author responsible for submission and editorial communication.

## Funding

No funding was received for this manuscript.

## Ethics Statement

Approval was obtained from the Institutional Review Board of the Palestinian Red Crescent Society Hospital, Hebron, Palestine. The committee does not assign formal approval numbers, as is common practice in many institutions in our region. Verbal informed consent to participate was obtained from both the patient and his father before inclusion in this case report.

## Consent

Verbal informed consent for publication of this case report and any accompanying images was obtained from both the patient and his father.

## Conflicts of Interest

The authors declare no conflicts of interest.

## Supporting information


**Supporting Information 1** Additional supporting information can be found online in the Supporting Information section. CARE Checklist (Done‐English‐2013.pdf). The supporting file includes the completed CARE (CAse REport) checklist in accordance with the CARE 2013 reporting guidelines. This checklist confirms that all essential components of a high‐quality case report have been appropriately addressed in the manuscript, thereby enhancing transparency, completeness, and reporting accuracy.

## Data Availability

The data that support the findings of this study are available on request from the corresponding author. The data are not publicly available due to privacy or ethical restrictions.
